# High Respiratory Tract Infection Rate in Patients With Familial Mediterranean Fever

**DOI:** 10.7759/cureus.29700

**Published:** 2022-09-28

**Authors:** Melih Hangül, Sema Akman, Mustafa Koyun, Sadıka H Akbas

**Affiliations:** 1 Pediatric Pulmonology, Akdeniz University Faculty of Medicine, Antalya, TUR; 2 Pediatric Nephrology, Akdeniz University Faculty of Medicine, Antalya, TUR; 3 Biochemistry, Akdeniz University Faculty of Medicine, Antalya, TUR

**Keywords:** pediatric infectious disease, pediatric rheumatology, mannose-binding lectin, respiratory tract infection, familial mediterranean fever

## Abstract

Background

Familial Mediterranean fever (FMF) is a systemic autoinflammatory disease genetically transmitted with the autosomal recessive trait. Although the pathogenesis is not certain, it is known that there is a deficiency in the regulation of the natural immune system. In this study, we aimed to search whether patients with FMF are predisposed to respiratory tract infections and whether mannose-binding lectin (MBL), as a natural immune system member, contributes to it.

Methods

Fifty FMF patients and 20 control groups were enrolled in the study. First, the frequencies of upper respiratory tract infection (URTI) within the previous year of both patient and control groups were evaluated retrospectively. Then, both groups were followed up for URTI for one year after starting the study. The patient's immunoglobulin A (IgA), IgM, IgG, C-reactive protein (CRP), hemogram parameters, and mannose-binding lectin were evaluated.

Results

The median frequency of annual URTI with a retrospective evaluation of patients was higher than that of the control group. Also, the median frequency of URTI with the prospective evaluation of patients with FMF was higher than the control group. The rate of patients with low serum IgG levels was higher in the patient group than in the control group. However, serum IgG levels of FMF patients with frequent URTI were not different from those without. The median MBL levels of both groups were similar (1312 vs. 1534 ng/ml for the patient and control group, respectively). The rate of patients having low serum MBL levels was also similar between the groups.

Conclusions

In the present study, we found that patients with FMF had more URTI than healthy controls. However, the underlying cause is not fully explained. There is a need for further studies with a higher number of patients evaluating URTI in patients with FMF.

## Introduction

Familial Mediterranean fever (FMF) is a systemic autoinflammatory disease, genetically transmitted with the autosomal recessive trait, characterized by repetitive and self-limiting attacks of fever, pleuritis, synovitis, peritonitis, and skin rashes at irregular intervals [1]. Protein pyrin, a 781 amino acid immunoregulatory molecule, is produced by the MEFV gene. It interacts with caspase-1 and other elements of the inflammasome to control interleukin-1 (IL-1) production, playing a significant function in the innate immune system. Most of the pyrin mutations are found in the protein's C-terminal B20.2 domain, which is where pyrin directly binds pro-caspase-1 and inhibits IL-1 activations. As a result, the inhibitory action of pyrin on caspase-1 is reduced in FMF patients with mutant pyrin. This event leads to inflammation in these people. Although the pathophysiology of FMF is not completely explained, it is known that there is a deficiency in the regulation of the natural immune system [2]. Autoinflammatory diseases are now categorized within primary immunodeficiency diseases [3]. No data exist on whether FMF patients have other accompanying defects in regulating the natural immune system. Mannose-binding lectin (MBL) is an essential mediator of innate immunity that can activate an antibody-independent complement system. It also has a significant role in the opsonization and phagocytosis of bacteria [4]. Over the past 10 years, the increasing number of researches highlighting the part of MBL in the natural immune system and especially uncovering the relevance between some diseases with MBL2 gene polymorphisms has gotten MBL more attention. It has been shown that MBL gene polymorphisms and low MBL levels increase the risks of some infectious diseases and contribute to the formation of autoimmune diseases such as systemic lupus erythematosus, Sjogren syndrome, and Behcet’sdisease [5-8]. MBL deficiency is significant since it is common in some immune deficiencies, increasing the risk of respiratory tract infections [9].

This study aimed to search whether patients with FMF have a predisposition for respiratory tract infections and whether MBL, as a natural immune system member, contributes to it.

## Materials and methods

Fifty patients diagnosed with FMF according to Tel Hashomer criteria at our department were enrolled [10]. Patients younger than three years old were excluded. Patients with an acute attack were not evaluated. There were no complications related to FMF in our patients. Genetic analysis of all patients was performed. The genetic analysis of four patients was normal. In other patients, the most common mutations were M694V (60.8%), E148Q (19.5%), R202Q (19.5%), M680I (17.3%), V726A (10.8%), E230K (2.17%), E230Q (2.17%) and P110C (2.17%). The control group was selected from 20 healthy, age and sex-matched children without FMF in their relatives or without any symptom or sign of FMF. The study was performed after approval from Akdeniz University Clinical Research Ethics Committee. All study procedures were performed by the ethical principles of the 1964 Declaration of Helsinki.

First, the frequencies of upper respiratory tract infection (URTI) within the previous year of both patient and control groups were evaluated retrospectively. Then, both groups were followed up for URTI for one year after. If they had any symptoms of URTI within this period, they applied and were examined, and then the frequency of URTI was recorded. An information survey form was prepared for patients, including age, gender, drug use for URTI, response to treatment, annual upper respiratory tract infection rate, and history of acute rheumatic fever.

Upper respiratory tract infection is defined as diagnosis through the patient's medical records and then history to distinguish between recurrent and non-recurrent URTI. Diagnosis from the same physician if the patient has one or more URTIs, including rhinitis, sinusitis, pharyngitis, and one or more symptoms (cough, sore throat, rhinorrhea, nasal congestion, hyposmia, or anosmia) that meet the criteria for URTI. Patients with eight or more URTIs per year were defined as frequent URTIs [11-14].

They were divided into two groups based on the colchicine dose as lower or higher than 0.05 mg/kg/day [15]. Patients with a history of allergies and gastroesophageal reflux were excluded from the study.

Blood and serum samples were taken from patients and control groups. The serum section was separated by centrifuging the tube for biochemical and immunological parameters, and the sample was kept at -80ᵒC until the study began. Since whole blood, sedimentation, and urine tests could not be held, they were run simultaneously with blood collection. Pediatric immunoglobulin A (IgA), IgM, IgG, C-reactive protein (CRP), sedimentation, and MBL were examined in serum samples. Pediatric IgA, M, and G levels were evaluated according to age [16,17].

Human MBL Quantikine ELISA (enzyme immunoassay) kit (R&D System Inc., Minneapolis, USA/DMBL00) was used to determine MBL serum levels. This assay employs the quantitative sandwich enzyme immunoassay technique. A monoclonal antibody specific for human MBL has been pre-coated onto a microplate. Standards and samples were pipetted into the wells, and the immobilized antibody bound any MBL present. After washing away any unbound substances, an enzyme-linked monoclonal antibody specific to MBL was added to the wells. After a wash, a substrate solution was added to the wells to remove any unbound antibody-enzyme reagent. The color developed in proportion to the amount of MBL bound in the initial step. The color development was stopped, and the intensity of the color was measured; 600 ng/ml was accepted as the cutoff value for MBL deficiency [18].

Statistical analysis

Data of the patient and control groups were recorded using SPSS 22.0 Windows software package (IBM Corp. Armonk, NY). Shapiro-Wilk and Kolmogorov-Smirnov tests were used for tests of normality. Descriptive statistics were presented as frequency, percentage, mean with standard deviation (SD), and median with minimum-maximum values. Fisher's exact test or Pearson's chi-square test analyzed the relationships between categorical variables. Intergroup comparisons of numerical data matching normal distribution in independent groups by the student t-test. The Mann-Whitney U test evaluated variables that did not fit into the normal distribution.

## Results

Demographic features

The mean age of the patient and control group were similar (11.0 ± 4.6 and 11.9 ± 4.2 years, respectively) (Table 1). The male and female ratio was 1.9 and 0.5 for the patient and control groups, respectively. The mean age at diagnosis of FMF was 7.9 ± 4.6 years. The mean follow-up period was 4.0 ± 2.2 years (Table 1).

**Table 1:** The evaluation of low immunoglobulin, MBL levels, serum, and frequency of URTI CRP: C-reactive protein, Ig: immunoglobulin, MBL: mannose-binding lectin, URTI: upper respiratory in infection, *Low Ig level for age, M: male, F: female

	Patient Group (n=50)	Control Group (n=20)	p
Age	11.0 ± 4.6	11.9 ± 4.2	0.543
Gender (M/F)	33/17 (66%/34%)	7/13 (35%/65%)	0.06
Hemoglobin (g/dl)	12.5180±1.31	13.1400±1.40	0.025
Leukocyte ( mm³)	7350.8± 2062	8231± 2233	0.06
Neutrophil (mm³)	3656.3±1350	4624±2201	0.10
CRP (mg/dl)	0.3872±1.1	0.1135±0.11	0.71
Serum IgA (mg/dl)	144.154±73	167.995±87	0.341
Low serum IgA *	11 (22%)	1 (5%)	0,880
Serum Ig G (mg/dl)	913.9±222	979.55±244	0.22
Low serum IgG*	23 (46%)	2 (10%)	0.005
Serum IgM (mg/dl)	108.354±49	114.26±49	0.711
Low serum Ig M *	14 (28%)	4 (20%)	0.489
MBL (ng/ml) (min-max)	1312 (356-4036)	1534 (296-4036)	0.79
Serum MBL level (<600 ng/ml)	10 (20%)	2 (10%)	0.336
Retrospective frequency of URTI (min-max)	4.56 (min: 1 max: 13)	1.85 (min: 0 max: 4)	0.001
Prospective Frequency of URTI (min-max)	4.2 (min: 1- max: 12)	1.85 (min: 0 max: 4)	0.001

Clinical features

The median frequency of annual URTI with a retrospective evaluation of patients was higher than that of the control group (4.56 (1-13) vs.1.85 (0-4) p= 0.001). Also, the median frequency of URTI with a prospective evaluation of patients with FMF was higher than the control group (4.2 (1-12 vs. 1.85 (0-4) p = 0.001) (Table 1). Besides, 10 (20%) of the patients with FMF had frequent URTI, whereas none had URTI in the control group (p = 0.031) (Table 1).

The colchicine dose was under 0.05 mg/kg/day in 41 patients (82.0%). The frequency of URTI was similar in patients having colchicine doses over and under 0.05 mg/kg/day (Table 2).

**Table 2:** Laboratory findings of frequent URTI and non-frequent URTI MBL: mannose-binding lectin, URTI: upper respiratory in infection, Ig: immunoglobulin, *Low Ig for age

	Frequent URTI (n:10 / 20%)	Non-frequent URTI (n:40 / 80%)	p
Age	7 (4-15)	13.5 (5-18)	0.01
Hemoglobin (g/dl)	12.1±0.5	12.6±1.40	0.33
Leukocyte (mm³)	8888± 2301	6966± 1835	0.06
Neutrophil (mm³)	3090±1772	3593±1242	0.51
Lymphocyte(mm³)	3980±1102	3392±944	0.47
CRP (mg/dl)	0.38±0.4	0.39±1.2	0.96
Serum Ig A (mg/dl)	115±92	146±69	0.64
Low serum Ig A *	3 (27.3%)	8 (72.7%)	0.465
Serum IgG (mg/dl)	817±195	938±223	0.22
Low serum Ig G*	5(21.7%)	18 (78.3%)	0.777
Serum IgM (mg/dl)	116±49	106±49	0.711
Low serum Ig M *	2 (14.3 %)	12 (85.7%)	0.529
MBL (ng/ml) (min-max)	3336 (356-4036)	1074 (336-4036)	0.53
Serum MBL level (<600 ng/ml)	2 (20%)	8 (80%)	0.336
Colchicine dose >0.05	3 (33.3%)	6 (66.7%)	0.421
Colchicine dose <0.05	7 (17.1%)	34 (82.9%)	0.523

Laboratory results

The mean leukocyte, serum IgA, IgG, and IgM values were similar in the patient and control groups (Table 1). The Ig levels of the patient and control groups were classified as normal or low based on their age-appropriate values. The two groups were compared, and there was no statistically significant difference (Table 1). However, the mean hemoglobin levels of the patient group were lower than the control group (p = 0.025) (Table 1). One of the patients (2%) in the FMF group had neutropenia, and one (2%) patient had lymphopenia. The median MBL levels of both groups were similar (1312 vs. 1534 ng/ml, for the patient and control group, respectively). Also, the rate of patients having low serum MBL levels (cut-off <600 ng/ml; 18) was also similar between the groups (20% vs. 10% for patient and control group, respectively) (Table 1). The rate of patients with low serum IgG levels was higher in the patient than in the control group (46% vs. 10%, p=0,005) (Table 1). However, the serum IgG levels of FMF patients with frequent URTI were not different from those without (Table 2). The rate of patients with low serum IgA and IgM levels was similar between the two groups (Table 2). The demographic, clinical, and laboratory data of patients with frequent URTI and non-frequent URTI were given in Table 2. There was no correlation between the frequency of URTI and serum IgA, IgG, IgM, and MBL levels (p=0.177 rho= -0.163, p=0.850 rho= -0.246, p=0.376 rho= 0.128 p=0.907rho= 0.017, respectively).

## Discussion

In the present study, we found that patients with FMF have frequent URTI compared to the normal population. It may be due to the insufficiency of the regulation of the natural immune system or the immune deficiency in the other systems that may accompany the pathogenesis of the disease. Antibody synthesis disorders are the most common primary immunodeficiency diseases among all primary immunodeficiencies; the disease spectrum ranges widely. On one spectrum, there exist patients with a mild clinical course and slightly lower levels of immunoglobulins. In contrast, on the other spectrum, severe diseases exist such as agammaglobulinemia, where all immunoglobulins are low [19]. Our study evaluated the serum IgA, IgG, and IgM levels of patients with FMF. We found normal serum IgA levels in patients with FMF compared to the control group. Isaacs et al. reported that children with low IgA levels had more frequent URTI; however, there was no statistically significant correlation between serum IgA, IgG, and frequent URTI in their study [20]. Similarly, no significant correlation between low levels of serum IgA and IgG with frequent URTI was found in our study.

Further studies evaluating secretory IgA levels responsible for mucosal immunity may give better results. We found that nearly half of the patients with FMF had low serum IgG levels; however, the patients with low serum IgG levels did not have frequent URTI. Inocencio et al. reported a case of a patient with FMF with frequent URTI having normal levels of IgG, IgA, and IgM; however, in further Ig subgroup analysis, IgG3 deficiency was detected in this patient, and frequent URTI decreased after starting intravenous immunoglobulin treatment [21]. There may be a deficiency in the IgG subgroup of our patients; unfortunately, we did not study IgG subgroups. For this reason, further studies evaluating IgG subgroups would be helpful in FMF patients. Tsinti et al. reported that a patient with FMF who had frequent URTI had lymphopenia and low serum IgG, IgA, and IgM levels [22]; the laboratory values improved after switching colchicine to canakinumab.

We investigated if low levels of IgG may be due to colchicine in FMF patients. and divided the patients into two groups - having low and high-dose colchicine use to evaluate the effect of colchicine on frequent URTI and found no effect of colchicine dose on developing frequent URTI. So, we invalidated the hypothesis that frequent URTI was due to an immune deficiency resulting from the side effect of a high dose of colchicine. Lymphopenia and neutropenia may develop in patients receiving colchicine, which returned to normal levels after reducing the drug dose [23]. Similar effects are observed in overdosing [24]. In a study by Yalçinkaya et al., transient leukopenia was reported due to colchicine treatment in children with FMF [15]. In our research, neutropenia and lymphopenia-induced immune deficiency leading to frequent infections were not detected in any patient. However, in our study, there were patients with transient leukopenia.

In recent years, the serum level of mannose-binding lectin, an essential protein of the lectin pathway was found to differ according to genetic polymorphism in the activation of the complement system. It has been shown that low serum levels of MBL are predisposed to infectious diseases [25,26]. MBL deficiency has particular importance because it increases the incidence of respiratory tract infections and is a common immunodeficiency [9]. As we know, this is the first study examining the relationship between MBL levels of FMF patients compared to healthy controls and MBL serum levels of FMF patients with frequent URTI and those without. We found that serum MBL levels of patients with FMF and healthy controls were. A few studies exist examining the relationship between frequent URTI and MBL in children other than FMF. As a result of these studies, there are conflicting results regarding MBL and frequent URTI [18,27,28]. Cedzynski et al. divided 335 patients between the ages of one and 16 who had recurrent respiratory tract infections into four groups - having no immunodeficiency, cellular immunodeficiency, humoral immunodeficiency, and allergy [18]. They found that low levels of MBL were a risk factor for recurrent respiratory tract infection, especially in those with humoral immune deficiency. In their cohort study, Thorarinsdottir et al. claimed that MBL is not an independent risk factor for respiratory infections in children aged two to four years and emphasized that accompanying Ig deficiencies triggered diseases [27]. Aittoinemi et al. found that serum MBL levels of patients with frequent respiratory system infections were similar to healthy controls [28]. As a result, frequent respiratory tract infections cannot be explained by low serum MBL levels. A study with adult patients with FMF having MBL gene R52CC>T and G54DG>A polymorphisms showed that the frequency was not different from healthy individuals [29].

Limitations

The current study had several limitations. The patient and control populations were quite smaller; further prospective analyses with a larger population are needed to confirm. Additionally, our research was unable to analyze all immunological markers. Individuals in this category who often have upper respiratory infections may determine why by examining other immunological markers such as Ig and lymphocyte subgroups.

## Conclusions

In the present study, we found that patients with FMF had more URTI than healthy controls. However, the underlying cause is not fully explained. We also found that nearly half of the patients with FMF had low serum IgG levels. More frequent URTI in FMF patients suggests another immunodeficiency disorder that may be associated with the immune regulation disorder of the pathogenesis of the disease. There is a need for further studies with a higher number of patients evaluating URTI in patients with FMF.
